# Patient Complexity, Social Factors, and Hospitalization Outcomes at Academic and Community Hospitals

**DOI:** 10.1001/jamanetworkopen.2024.54745

**Published:** 2025-01-15

**Authors:** Michael Colacci, Anne Loffler, Surain Bala Roberts, Sharon Straus, Amol A. Verma, Fahad Razak

**Affiliations:** 1 Li Ka Shing Knowledge Institute, St Michael’s Hospital, Toronto, Ontario, Canada; 2 Department of Medicine, University of Toronto, Toronto, Ontario, Canada; 3 Institute of Health Policy, Management, and Evaluation, University of Toronto, Toronto, Ontario, Canada

## Abstract

**Importance:**

There have been limited evaluations of the patients treated at academic and community hospitals. Understanding differences between academic and community hospitals has relevance for the design of clinical models of care, remuneration for clinical services, and health professional training programs.

**Objective:**

To evaluate differences in complexity and clinical outcomes between patients admitted to general medical wards at academic and community hospitals.

**Design, Setting, and Participants:**

This retrospective cohort study of patients admitted to general medicine at 28 hospitals in Ontario, Canada, was conducted between April 2015 and December 2021. All patients admitted to or discharged from general medicine during the study period who were older than 18 years were included. Data analysis occurred between February 2023 and June 2024.

**Exposures:**

Patient admission to a general medicine inpatient service at an academic or community hospital.

**Main Outcomes and Measures:**

Demographic and clinical characteristics (age, sex, modified Laboratory-based Acute Physiology Score [mLAPS], discharge diagnosis, Charlson Comorbidity Index, frailty risk score, and disability), social factors (neighborhood-level markers of income, material deprivation, immigrant status, and racial and ethnic minority status) and clinical outcomes and processes (patient volume per physician, in-hospital mortality, length of stay, readmission rates, and intensive care unit [ICU] admission rates).

**Results:**

There were 947 070 admissions, including 609 696 at 17 community hospitals (median [IQR] age, 73 [58-84] years) and 337 374 at 11 academic hospitals (median [IQR] age, 70 [56-82] years). Baseline clinical characteristics were similar at community and academic hospitals, including female sex (307 381 [50.4%] vs 168 033 [49.8%]; standardized mean difference [SMD] = 0.012), median (IQR) mLAPS (21 [11-36] vs 21 [10-34]; SMD = 0.001), and Charlson Comorbidity Index score of 2 or greater (182 171 [29.9%] vs 105 502 [31.3%]; SMD = 0.038). Social characteristics, including income, education, and neighborhood proportion of racial and ethnic minority and immigrant residents were also similar. The number of unique discharge diagnoses was similar at academic and community hospitals. Patient volumes per attending physician were higher at academic hospitals (median [IQR] daily census, 20 [19-22] vs 17 [15-19]; SMD = 1.086). After multivariable regression adjusting for baseline factors, mortality (adjusted odds ratio [aOR], 0.96; 95% CI, 0.78 to 1.17), ICU admission rate (aOR, 1.20; 95% CI, 0.80 to 1.79) and length of stay (β =  −0.001; 95% CI, −0.10 to 0.10) were not significantly different, while 7-day readmission (aOR, 1.25; 95% CI, 1.10 to 1.43) and 30-day readmission (aOR, 1.25; 95% CI, 1.10 to 1.42) were significantly higher at academic hospitals than community hospitals.

**Conclusions and Relevance:**

In this cohort study, patients admitted to general medicine at academic and community hospitals had similar baseline clinical characteristics and generally similar clinical outcomes, with greater readmission rates in academic hospitals. These findings suggest that the patient case mix in general internal medicine that trainees would be exposed to during their residency training at academic hospitals is largely representative of the case mix they would encounter at community hospitals, and has important implications for health services planning and funding.

## Key Points

**Question:**

How do academic and community hospitals compare with respect to patient baseline characteristics, social factors, staffing ratios, and hospitalization outcomes?

**Findings:**

In this cohort study of 947 070 general medicine encounters at 28 academic and community hospitals in Ontario, Canada, patients had similar baseline clinical and social characteristics. Patient volumes per physician and patient readmission rates were higher at academic hospitals, whereas hospital mortality and length of stay were similar.

**Meaning:**

In this cohort study, the patients treated at academic and community hospitals and corresponding clinical outcomes were generally similar, which has implications for both health professional training programs and health services planning.

## Teaser

This cohort study compares differences in patient case mix and clinical outcomes between academic and community hospitals among patients admitted to general medicine in Ontario, Canada.

## Introduction

Academic and community hospitals both provide essential health services to the population. Among their many health-related activities, academic hospitals provide patient care, educate future health care professionals, and advance health research,^1,2^ while community hospitals provide the largest proportion of the population’s medical care, serve the greatest proportion of municipalities,^3^ and play a growing role in education and research.^4^ Despite differences in their priorities and roles, it is unclear if academic and community hospitals care for different types of patients. These potential differences are important for several reasons. First, academic medical centers have faced difficulty in recruiting and retaining faculty as well as maintaining accreditation, and increased patient complexity compared with community sites has been proposed as one potential reason for this (in addition to the competing demands of teaching and other academic responsibilities).^1,5,6,7,8,9,10,11,12,13,14,15,16,17,18,19^ Second, it is unclear whether the patients seen by medical trainees during their residency at academic hospitals are representative of the patients they may later see in practice at community hospitals, which has implications for preparedness to enter independent practice. Third, the characteristics of patients cared for at academic and community hospitals have important implications for health services planning and funding. This includes ensuring appropriate diagnostic testing and subspecialty physician availability to address patient care needs.

We investigated differences in patient characteristics and clinical outcomes between academic and community hospitals in Ontario, Canada. We specifically focused on patients treated by general medicine services because they account for the largest proportion of hospital admissions from the emergency department (>40% in most cases), and the growing volume of general medicine patients has been a major source of capacity pressure due to rising rates of comorbidity and an aging population.^20,21^ Furthermore, general medicine services are a central site of training in hospital-based care.

## Methods

### Study Design, Setting, and Participants

We conducted a retrospective cohort study of general medicine patients at 28 hospitals in Ontario, Canada, between April 2015 and December 2021 that are part of the General Medicine Inpatient Initiative (GEMINI), a hospital-based research network.^21^ Our study was approved by the research ethics boards for all included hospitals; the requirement for written informed consent was waived by the research ethics board given the use of deidentified patient data. Ontario is the largest single-payer health system in North America with a population of 14.2 million people.^22,23^ Hospital services are insured for all residents of Ontario at no charge to patients. This study was reported in accordance with Strengthening the Reporting of Observational Studies in Epidemiology (STROBE) reporting guideline.^24^

All patients admitted to or discharged from general medicine during the study period who were older than 18 years were included. We included both emergency department admissions and direct admissions. No exclusion criteria were applied. Patients were followed from the date of hospital admission to the latest of either in-hospital death, hospital discharge, or readmission within 30 days to a hospital within the GEMINI database. The GEMINI database includes administrative data (at both the patient and clinician level) and clinical data for all patients admitted to, or discharged from, the medical units at the affiliated sites.^21^ Hospitals in the GEMINI network encompass more than 60% of medical hospitalizations in Ontario and include academic hospitals affiliated with 5 of the province’s 6 independent medical schools.

### Data Sources

Administrative and clinical data were extracted from each patient’s electronic health records through previously described methods.^21,25^ In brief, deidentified electronic health records are transferred from participating hospitals to a central site where they are processed and standardized. Data include diagnoses, transfer of care between hospital services, investigations (laboratory, radiologic, and microbiologic), vital signs, and medications prescribed in hospital. Data undergo routine quality assessments and have been found to have an accuracy of 98% to 100% across variables in comparison with manual medical record review.^25^ Data from patients’ electronic medical records were linked to data reported by hospitals for the Canadian Institute for Health Information (CIHI) Discharge Abstract Database and the National Ambulatory Care Reporting System.^26,27^ For the majority of social characteristics (education, income, racial and ethnic minority status, immigrant status, and material deprivation), individual patient-level data are not collected in Canadian hospitals. As such, we obtained neighborhood-level information by linking patient’s postal codes to 2016 census data and used this to calculate the components of the Ontario Marginalization Index, a measure of neighborhood demographic characteristics including material resources, racial and ethnic minority and newcomer populations, age and labor force, and residential structure.^28^

### Measures and Definitions

Hospitals were defined as academic if they were fully affiliated with a university and medical school and as community hospitals otherwise.^22^ There were 2 primary differences between academic and community hospitals of relevance for this study. First, academic hospitals operated in a tiered care structure where the care team was composed of medical students, residents, clinical fellows, and attending physicians. In contrast, at community hospitals, care was typically provided directly by attending physicians. Second, the ratio of clinical to nonclinical work of attending physicians was different at academic and community hospitals. At academic hospitals, attending physicians had a dual job description that included a relatively fixed ratio of clinical to nonclinical work, with academic clinicians spending up to 80% of their time performing nonclinical work (teaching and education, quality improvement, or research and administration). In contrast, attending physicians at community hospitals typically had a job description where the majority of their time was spent performing clinical work. Other important factors, such as availability of interdisciplinary health services (eg, social work, case management, and physiotherapy or occupational therapy) were generally similar at the included academic and community hospitals. These differences are relatively similar to those in the US, where an academic medical center is fully affiliated and integrated with a medical school. At each hospital, we measured the following variables for each general medicine admission: (1) baseline clinical characteristics, including whether the patient was admitted from a long term care (LTC) facility, age, sex, Charlson Comorbidity Index, frailty score (Hospital Frailty Risk Measure), disability, modified Laboratory-Based Acute Physiology Score (mLAPS), and most responsible discharge diagnosis; (2) social characteristics, including neighborhood level markers of income, material deprivation, immigrant status, and racial and ethnic minority concentration; and (3) clinical processes of care and outcomes, including median daily patient census per general medicine attending physician, in-hospital mortality, length of stay, readmission rates, intensive care unit (ICU) admission rates, and proportion of admissions where patients were designated to an alternate level of care (ALC; defined as patients who occupy an inpatient bed but do not require the intensity of inpatient services).^28,29,30,31,32,33^ The mLAPS is a validated predictor of in-hospital mortality risk, calculated using 13 laboratory tests completed up until 24 hours after admission.^31^ Disability was a binary individual-level variable defined using established *International Statistical Classification of Diseases and Related Health Problems, Tenth Revision (ICD-10)* algorithms for physical, sensory, and cognitive disability.^34^ The median daily patient census per attending general medicine physician was calculated as a measure of patient volume per physician. This was calculated by dividing the total general medicine daily census (calculated at 8:00 am local time) by the median number of ward attending physicians working each day in a hospital. Length of stay (LOS) was modeled as a continuous variable. Because LOS was heavily right-skewed, we log-transformed LOS prior to model fitting. To facilitate interpretation, model estimates were transformed back to the original scale of LOS (days) for the reported results.

We also evaluated differences in case mix based on patients’ most responsible discharge diagnosis, which is defined in the CIHI Discharge Abstract Database as the diagnosis or condition that can be described as being most responsible for the patient’s stay in hospital.^27^ Diagnoses were coded using the *International Statistical Classification of Diseases and Related Health Problems, Tenth Revision, Canada* (*ICD-10-CA*) codes, and then categorized into clinically relevant categories using the clinical classification software refined (CCSR) (Healthcare Cost and Utilization Project).^35,36^ To quantify the breadth of conditions managed on general medical wards, we also report the number of unique CCSR diagnosis categories that accounted for 25%, 50%, 75%, 90%, and 100% of all patient hospitalizations at both academic and community sites. Readmissions were defined as return to any medical or intensive care service at a GEMINI hospital within 7 or 30 days of discharge from the initial episode of care. An episode of care refers to all contiguous inpatient hospitalizations to any medical or intensive care service. Episodes with discharge as death were excluded from the readmission denominator. Additionally, hospitalizations were not counted as readmissions if they were coded as elective.

### Missing Data

Laboratory data at some hospitals were missing for certain time periods (eFigure 1 in Supplement 1), resulting in missing mLAPS values for some hospital admissions. Admissions from time periods without any laboratory data availability (9.8%) were excluded from all analyses with mLAPS as an exposure or outcome variable. For time periods where laboratory data were available, if a patient had no laboratory tests in the 24 hours following admission to compute mLAPS (1.6% of admissions), scores were imputed with 0 given that the lack of laboratory tests typically implies that there was no indication for testing.

### Statistical Analysis

We first present descriptive statistics for baseline clinical characteristics, social characteristics, and clinical processes and outcomes in the academic and community hospital groups, reporting standardized mean differences (SMDs). SMDs of 0.1 or less were considered to demonstrate balance between groups.^37^ We then present statistical inference testing using generalized linear mixed-effects models to account for the fact that patients were clustered within hospitals, and hospitals presented very different sample sizes based on volume of care. Hospital affiliation (academic vs community) was modeled as a fixed effect and individual hospitals were modeled as random intercepts. For binary outcomes, odds ratios (ORs) are reported in addition to β coefficients to facilitate interpretation. To assess whether academic or community affiliation had a significant association with a given outcome variable, likelihood ratio tests were used to compare each model with a null model without affiliation as a fixed effect.

Additionally, we assessed the variance explained by the random effects (individual hospitals) compared with the variance explained by community or academic affiliation by reporting the intraclass coefficient (ICC) and (partial) marginal *R*^2^, respectively.^38^ A comparison of these 2 measures indicates how much of the variance observed across patients was due to variation across hospitals in general (regardless of university affiliation) as opposed to variation between academic vs community sites specifically. For baseline and social characteristics, we computed the unadjusted estimates. For clinical outcomes (LOS, ICU admission, in-hospital death, readmission, and ALC), we adjusted for age, sex, Charlson Comorbidity Index, mLAPS, LTC residence, and disability to account for differences in case mix that contribute to clinical outcomes. These variables were chosen based on prior literature, causal plausibility, and evaluation of variable collinearity.^29,39,40^ For clinical outcomes, we additionally present marginal effects to illustrate differences between academic vs community sites while controlling for baseline and social characteristics. Marginal effects were obtained using the mean value of each covariate as reference level. Patients who received palliative care (defined as most responsible discharge diagnosis Z51.5) were excluded from the analysis of in-hospital death, and a sensitivity analysis on mortality was completed without exclusion of patients receiving palliative care.^41^ Analysis was completed between February 2023 and June 2024. Fitting of generalized linear mixed-effects models was performed using the glmmTMB package in R version 4.2.2 (R Project for Statistical Computing). The threshold for statistical significance was a 2-sided* P* < .05. Please see the eMethods in Supplement 1 for more information on statistical analysis.

## Results

### Baseline Clinical Characteristics

The total cohort included 947 070 admissions, with 609 696 admissions at 17 community hospitals (median [IQR] age, 73 [58-84] years) and 337 374 admissions at 11 academic hospitals (median [IQR] age, 70 [56-82] years) (Table 1). The proportion of patients who were female was similar for both community and academic hospitals (307 381 individuals [50.4%] vs 168 033 [49.8%]; SMD = 0.012). Median (IQR) mLAPS was 21 (11-36) at community and 21 (10-34) at academic sites (SMD = 0.001). The proportions of individuals with a Charlson Comorbidity Index score of 2 or greater were similar across community and academic sites (182 171 [29.9%] vs 105 502 [31.3%]; SMD = 0.038). There were 85 010 patients (13.9%) with a disability at community hospitals and 49 825 (14.8%) at academic hospitals. The median (IQR) number of monthly admissions per site was 456 (350-571) at community hospitals and 428 (360-492) at academic hospitals, and the median (IQR) daily general medicine patient census was 125 (95-173) at community and 121 (102-138) at academic hospitals.

**Table 1.** Baseline Clinical Characteristics of Patients at Academic and Community Hospitals

Characteristic	Hospitalizations, No. (%)			SMD
	Overall (N = 947 070)	Community hospitals (n = 609 696)	Academic hospitals (n = 337 374)	
Hospital characteristics				
Hospitals, No.	28	17	11	NA
Monthly admissions per site, median (IQR)	440 (354-540)	456 (350-571)	428 (360-492)	0.267
Daily general medicine patient census, median (IQR)	125 (100-164)	125 (95-173)	121 (102-138)	0.869
Daily general medicine patient volume/physician, median (IQR)	19 (16-20)	17 (15-19)	20 (19-22)	1.086
Patient characteristics				
Age median (IQR), y	72 (57-84)	73 (58-84)	70 (56-82)	0.117
Sex				
Male	471 656 (49.8)	302 315 (49.6)	169 341 (50.2)	0.012
Female	475 414 (50.2)	307 381 (50.4)	168 033 (49.8)	
From long-term care home	90 478 (9.6)	64554 (10.6)	25924 (7.7)	0.101
Modified Laboratory-Based Acute Physiology Score, median (IQR)	21 (10-35)	21 (11-36)	21 (10-34)	0.001
Charlson Comorbidity Index score at admission				
0	488 696 (51.6)	318 717 (52.3)	169 979 (50.4)	0.038
1	170 701 (18.0)	108 808 (17.8)	61 893 (18.3)	
≥2	287 673 (30.4)	182 171 (29.9)	105 502 (31.3)	
Frailty score, median (IQR)	4.0 (2.0-5.0)	4.0 (2.0-5.0)	4.0 (2.0-5.0)	0.016
Presence of disability	134 835 (14.2)	85 010 (13.9)	49 825 (14.8)	0.024
Encounter characteristics				
10 Most frequent primary reasons for hospitalization^a^				
Heart failure	47 287 (5.0)	30 379 (5.0)	16 908 (5.0)	NA
Pneumonia	39 739 (4.2)	23 485 (3.9)	16 254 (4.8)	
Chronic obstructive pulmonary disease and bronchiectasis	37 348 (3.9)	19 598 (3.2)	17 750 (5.3)	
Urinary tract infections	35 322 (3.7)	22 973 (3.8)	12 349 (3.7)	
Neurocognitive disorders	29 536 (3.1)	19 551 (3.2)	9985 (3.0)	
Cerebral infarction	27 731 (2.9)	19 262 (3.2)	8469 (2.5)	
Septicemia	27 400 (2.9)	19 556 (3.2)	7844 (2.3)	
Diabetes with complication	22 002 (2.3)	13 643 (2.2)	8359 (2.5)	
Acute and unspecified kidney failure	21 144 (2.2)	14 657 (2.4)	6487 (1.9)	
Gastrointestinal hemorrhage	19 726 (2.1)	12 285 (2.0)	7441 (2.2)	

Abbreviations: NA, not applicable; SMD, standardized mean difference.

^a^ Based on most responsible discharge diagnosis based on Clinical Classifications Software Refined category.

The median (IQR) patient census per general medicine physician was higher at academic hospitals than community hospitals (20 [19-22] vs 17 IQR [15-19]; SMD = 1.086) (Table 1). Based on univariate mixed-effects models, patients at academic sites were less likely to be admitted from long term care than patients at community sites (OR, 0.69; 95% CI, 0.51-0.92; *P* = .02) (eTable 1 in Supplement 1). Other baseline characteristics did not show any statistically significant differences between academic vs community sites. For all baseline clinical characteristics, the variance explained by academic or community affiliation was less than 1% (mean [SD] marginal *R^2^* = 0.002 [0.003]) and was substantially smaller than the variance explained by hospital as a random effect (mean [SD] ICC = 0.032 [0.018]) (eTable 1 in Supplement 1).

### Social Characteristics

Overall, 929 165 patients (98.1%) had a postal code that was included in the 2016 census and linked to the Ontario Marginalization Index to obtain neighborhood-level data with no imbalance in missingness between community (9238 participants [1.5%]) and academic hospitals (8667 participants [2.6%]) (Table 2).^28^ Median (IQR) neighborhood-level after-tax income per person was CAD$45 400 (CAD$36 011-CAD$54 545) at community and CAD$44 099 (CAD$33 661-CAD$53 544) at academic hospitals (to convert to US dollars, multiply by 0.71091). The median (IQR) neighborhood-level percentage of people with postsecondary education was 54.05% (45.59%-62.46%) vs 56.00% (46.03%-66.27%), and the median (IQR) neighborhood proportion of racial and ethnic minority residents was 32.67% (12.96%-63.46%) vs 18.24% (6.41%-36.20%) at community and academic hospitals, respectively. The percentage of patients living in neighborhoods with the lowest material resources was 24.7% (147 521 participants) vs 27.6% (89 610 participants) at community and academic hospitals, respectively.

**Table 2.** Neighborhood-Level Aggregate Social Factors of Patients at Academic and Community Hospitals

Social factor	Hospitalizations, No (%)			SMD
	Overall (N = 947 070)	Community hospitals (n = 609 696)	Academic hospitals (n = 337 374)	
Missing census data	17 905 (1.9)	9238 (1.5)	8667 (2.6)	0.075
After-tax income/person, median (IQR), CAD$^a^	44 872 (35 243-54 181)	45 400 (36 011-54 545)	44 099 (33 661-53 544)	0.063
After-tax income/person, quintile				
1 (Highest)	240 487 (25.9)	144 888 (24.2)	95 599 (29.1)	0.125
2	178 589 (19.2)	119 890 (20.0)	58 699 (17.9)	
3	179 500 (19.3)	115 115 (19.2)	64 385 (19.6)	
4	172 775 (18.6)	117 079 (19.5)	55 696 (17.0)	
5 (Lowest)	156 642 (16.9)	102 784 (17.1)	53 858 (16.4)	
Postsecondary education, median (IQR), %	54.76 (45.71 to 63.49)	54.05 (45.59 to 62.46)	56.00 (46.03 to 66.27)	0.157
Racial and ethnic minority residents, median (IQR), %	25.49 (9.86 to 54.07)	32.67 (12.96 to 63.46)	18.24 (6.41 to 36.20)	0.534
Immigrant residents, median (IQR), %	34.55 (19.09 to 53.05)	40.99 (23.08 to 56.86)	25.56 (13.95 to 39.06)	0.632
Deprivation score, median (IQR)^b^	−0.08 (−0.66 to 0.71)	−0.09 (−0.64 to 0.66)	−0.05 (−0.69 to 0.83)	0.051
Material resources, quintile				
5 (Highest)	169 564 (18.4)	102 951 (17.2)	66 613 (20.6)	0.138
4	163 640 (17.8)	110 751 (18.5)	52 889 (16.3)	
3	160 624 (17.4)	111 164 (18.6)	49 460 (15.3)	
2	190 620 (20.7)	125 052 (20.9)	65 568 (20.2)	
1 (Lowest)	237 131 (25.7)	147 521 (24.7)	89 610 (27.6)	

Abbreviation: SMD, standardized mean difference.

^a^ To convert to US dollars, multiply by 0.71091.

^b^ Factor score from the Ontario Marginalization Index; higher values correspond to higher levels of deprivation.

In the univariable mixed-effects models for hospital affiliation, there were no neighborhood social factors that were significantly different at community and academic hospitals (Table 2 and eTable 2 in Supplement 1). This was also true for social factors with large absolute differences in the median values, including proportion of racial and ethnic minority and immigrant residents because there was significant variation within both community and academic hospitals and correspondingly a broad IQR. As with baseline characteristics, community or academic affiliation explained a negligible amount of variance observed in all social factors (mean [SD] marginal *R*^2^ = 0.004 [0.004]) and it accounted for less variance than the general variation across sites regardless of affiliation (mean [SD] ICC = 0.133 [0.108]) (eTable 2 in Supplement 1).

### Discharge Diagnosis

The distribution of discharge diagnoses was similar at community and academic hospitals (eFigure 2 in Supplement 1). The most common conditions at academic and community hospitals were heart failure (16 908 hospitalizations [5.0%] in academic hospitals and 30 379 hospitalizations [5.0%] in community hospitals), pneumonia (16 254 hospitalizations [4.8%] in academic hospitals and 23 485 hospitalizations [3.9%] in community hospitals), and urinary tract infection (12 349 hospitalizations [3.7%] in academic hospitals and 22 973 hospitalizations [3.8%] in community hospitals). The number of unique CCSR diagnoses that accounted for 25%, 50%, 75%, and 100% of hospitalizations was similar at both sites, indicating a similar distribution in the variety of cases managed (eFigure 3 in Supplement 1). The total number of discharge diagnoses was 405 at community hospitals and 392 at academic hospitals.

### In-Hospital Outcomes

Unadjusted in-hospital mortality was 7.4% (45 183 individuals) at community and 6.4% (21 457 individuals) at academic hospitals (Table 3). Median (IQR) LOS was 4.9 (2.4-9.8) days at community hospitals vs 4.7 (2.4-9.8) days at academic hospitals; a total of 53 862 patients (8.8%) were admitted to the ICU at community hospitals and 39 694 patients (11.8%) were admitted to the ICU at academic hospitals. Seven-day readmission rates were 4.6% (24 484 readmissions) at community hospitals and 5.7% (17 108 readmissions) at academic hospitals. The proportion of patients who had an ALC designation during their hospitalization was 62 785 patients (11.1%) at community hospitals and 41 076 patients (13.8%) at academic hospitals.

**Table 3.** Hospitalization Outcomes of Patients at Academic and Community Hospitals

Outcome	Participants, No. (%)			SMD
	Overall (N = 947 070)	Community hospitals (n = 609 696)	Academic hospitals (n = 337 374)	
In-hospital mortality	66 640 (7.0)	45 183 (7.4)	21457 (6.4)	0.042
Length of stay, median (IQR), d	4.8 (2.4-9.8)	4.9 (2.4-9.8)	4.7 (2.4-9.8)	0.021
Intensive care unit admission	93 556 (9.9)	53 862 (8.8)	39 694 (11.8)	0.097
7-d Readmission	41 592 (5.0)	24 484 (4.6)	17 108 (5.7)	0.049
30-d Readmission	115 233 (14.1)	68 636 (13.1)	46 597 (15.7)	0.074
Hospitalizations with alternate level of care	103 861 (12.1)	62 785 (11.1)	41 076 (13.8)	0.080

Abbreviation: SMD, standardized mean difference.

After adjusting for patient baseline factors, there were no significant differences between academic and community sites for in-hospital mortality (adjusted OR [aOR], 0.96; 95% CI, 0.78 to 1.17), LOS (β = −0.001; 95% CI, −0.10 to 0.10), ICU admission rate (aOR, 1.20; 95% CI, 0.80 to 1.79), or proportion of ALC admissions (aOR, 0.98; 95% CI, 0.22 to 4.34) (Table 4 and Figure). Academic hospitals had greater 7-day readmission rates (aOR, 1.25; 95% CI, 1.10 to 1.43) and 30-day readmission rates (aOR, 1.25; 95% CI, 1.10-1.42) than community hospitals (Table 4). Across all clinical outcomes, community or academic affiliation explained a negligible amount of variance (mean [SD] partial marginal *R^2^* = 0.002 [0.002]) compared with the variance explained by hospital as a random effect (mean [SD] ICC = 0.111 [0.210]) and the variance explained by all other covariates (mean [SD] marginal *R^2^* = 0.123 [0.111] for models without affiliation as a fixed effect). There was no significant difference in mortality in the sensitivity analysis where palliative deaths were included.

**Table 4.** Generalized Linear Mixed-Effects Model for Hospitalization Outcomes

Clinical outcome	OR (95% CI)^a^	*P* value	Intraclass coefficient	Marginal *R*^2 ^for affiliation
In-hospital mortality	0.96 (0.78 to 1.17)	.68	0.021	<0.001
Length of stay	β = 0.001 (95% CI, −0.10 to 0.10)^b^	.98	0.014	<0.001
Intensive care unit admission	1.20 (0.80 to 1.79)	.38	0.078	0.003
7-d Readmission	1.25 (1.10 to 1.43)	.002^c^	0.009	0.004
30-d Readmission	1.25 (1.10 to 1.42)	.001^c^	0.008	0.004
Alternate level of care admission	0.98 (0.22 to 4.34)	.98	0.537	<0.001

Abbreviation: OR, odds ratio.

^a^ β coefficients are shown for continuous variables and ORs for binary variables.

^b^ β for affiliation.

^c^ Statistically significant.

**Figure.**
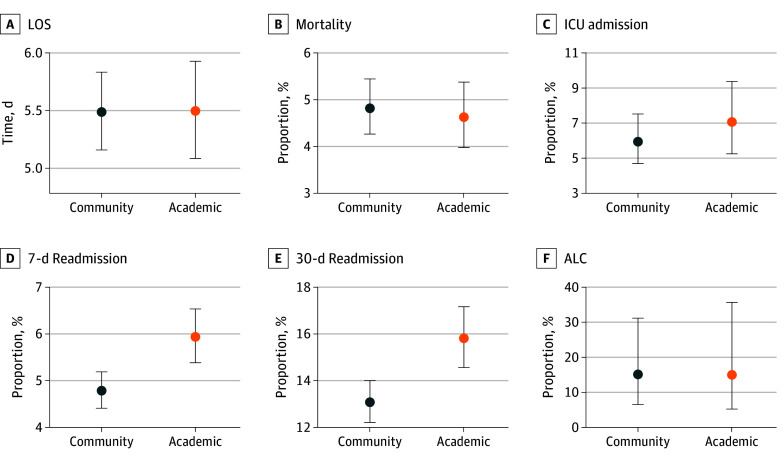
Marginal Effects for Clinical Outcomes Mean clinical outcomes at community (blue) vs academic (orange) hospitals were estimated based on a mixed-effects model controlling for baseline characteristics. Marginal effects were obtained using the mean value of each covariate as reference level. Error bars denote the SDs. ALC indicates alternate level of care; ICU, intensive care unit; LOS, length of stay.

## Discussion

This large cohort study of hospitals across all geographic regions of Ontario, Canada, found that general medicine patients at community and academic hospitals have similar baseline clinical complexity and social characteristics. There appears to be greater variation between hospitals in general than there is between academic and community hospitals specifically. At academic hospitals, the daily patient census for general medicine attending physicians was higher by approximately 3 patients at the median, and unplanned readmission rates were greater by approximately 1% to 3%, but there were no significant differences in mortality, ICU admission rates, or hospital LOS.

There have been few prior comparisons of general medicine patients at academic and community hospitals. One single center study^42^ that compared outcomes among patients admitted to a family medicine teaching service vs nonteaching hospitalist service found that LOS and cost of care were similar. Other comparisons of academic vs community hospitals have focused on outcomes for isolated disease entities or within cancer or subspecialty surgical care.^43,44,45,46,47,48,49,50,51^ These comparisons have identified increased patient complexity at academic centers, but have focused on nonemergent care for which patients were referred from an initial clinician, and are therefore only representative of a small sample of patients seen at academic and community hospitals. In contrast, general medicine accounts for roughly 40% of all hospital admissions, which typically occur on an emergent basis and comprise a broad range of diagnoses.^21^

Despite prior research, and potentially general perception, suggesting that patients at academic hospitals are more complex, we did not find this in our study.^51,52^ Patients at community hospitals were more likely to be admitted from an LTC home, but there were otherwise no meaningful differences in baseline characteristics or social factors. The absolute difference in median proportion of immigrants and racial and ethnic minorities between community and academic hospitals were a function of the significant range between hospitals and were not significant in the mixed-effect model. Furthermore, the difference in LTC residence between patients at academic and community hospitals was small, and there was greater variation among hospitals in general than there was between academic and community sites specifically. Attending physicians at academic medical centers did have a higher daily patient census, but this must be contextualized with the greater number of team members involved in general medicine services in academic centers, including resident physicians and medical students. When comparing clinical outcomes, we identified an increase in 7- and 30-day readmission rates at academic sites, which again varied to a greater degree between hospitals in general than between academic and community hospitals specifically. Possible contributors to this difference include unmeasured factors of clinical complexity (eg, residual confounding) or differences in care and discharge processes between academic and community hospitals.

Community hospitals in Canada face substantial sustainability challenges, including meeting the diverse needs of local communities with constrained resources, difficulty retaining and recruiting health care staff, substantial health inequities in rural areas, and challenges with access to specialty care.^53,54,55^ The sustainability of academic medical centers has also been called into question because they have faced difficulty in both recruiting and retaining faculty, as well as maintaining accreditation of their medical school and residency programs.^1,5,6,7,8,9,10,11,12,13,14,15,16,17,56,57,58^ These issues have been further exacerbated by the COVID-19 pandemic.^59,60^ Several reasons have been proposed as to why academic medical centers have faced difficulty. These include greater patient complexity in comparison with community hospitals, concurrently providing education to medical learners, and delivering on non–patient care-related academic responsibilities (eg, research, innovation, administration, and curriculum development).^13,14,15,18,19^ Our results suggest that differences in patient complexity between academic and community hospitals is not the primary factor responsible for the difficulties faced by different types of hospitals.

Patient case mix at academic hospitals is important for clinical trainees. Although residents complete much of their residency training at an academic center, less than one-half will work in an academic setting after completing residency.^61^ As such, it is important that the training provided in academic centers adequately prepares residents for future practice environments, including community hospitals. There has also been an increase in distributed medical education and community-based training opportunities.^62^ Our results suggest that the patient case mix in general internal medicine that trainees would be exposed to during their residency training at academic hospitals is largely representative of the case mix they would encounter at community hospitals, which supports the argument that both academic and community hospitals can provide excellent patient exposure.

### Limitations

Our study has several limitations. First, we dichotomized hospitals into academic and community based on whether they were fully affiliated with an academic site. There are a few partially affiliated community hospitals that operate with a hybrid model of care provision where medical trainees may be present. Even at these sites, the differences in job description between academic and community physicians is preserved (ie, proportion of time allocated to nonclinical work at hybrid sites and fully community sites is similar). Because the purpose of our analysis was to evaluate differences in patient characteristics that would ultimately be seen by academic and community physicians, we felt that this dichotomy of hospitals was appropriate. Furthermore, our results were unchanged in a sensitivity analysis where these hybrid sites were recategorized as academic hospitals (data not shown). Second, the GEMINI sample only includes large community hospitals. It is possible that there may be greater differences observed in smaller community hospitals. Relatedly, it is possible that a patient could have been readmitted to a hospital outside of the GEMINI network and not captured within this study. However, given the distribution of hospitals within the GEMINI network and hospital catchment areas, we believe this occurrence to be infrequent. Third, Canadian hospitals do not reliably collect important sociodemographic data at the patient level, and we therefore relied on neighborhood level markers. Fourth, some hospitals did not have data for the entire study period, and it is therefore possible that time trends affected the observed results, Fifth, it was outside of the scope of our work to examine differences in structures or processes of care between academic and community hospitals, including the number of interprofessional health staff, physician staffing models, or access to subspecialty resources. Therefore, our study was focused on exploring differences in patient populations, which is only one determinant of the overall experience of practicing or learning in an academic or community hospital. Sixth, although general medicine represents the largest single admitting service, accounting for approximately 40% of emergency admissions and 25% of hospital beds,^21^ we did not assess for differences in other clinical specialties. It is possible that there may be larger differences between academic and community hospitals in more subspecialized areas or in nonelective care.

As noted, hospital services in Ontario are universally insured for residents of the province. Therefore, our results may not generalize to health care settings without universal insurance for hospital care. Nevertheless, there are 2 implications from our study that are of direct relevance to these settings. First, when isolated from insurance status, our findings suggest that academic and community hospital affiliation does not independently affect patient complexity or case mix for emergent general medicine admissions. Second, and perhaps more importantly, for general medicine admissions, our findings suggest that hospital academic or community affiliation does not significantly impact important patient outcomes. We believe this finding to be less context-dependent and potentially generalizable to US settings.

## Conclusions

In this cohort study of patients admitted to medical wards at academic and community hospitals within a single payer health care system with universally insured hospital care, there were similar baseline clinical characteristics, social factors, and hospital outcomes. Academic hospitals had greater patient volume per physician and readmission rates. For all outcomes, there was greater variability between hospitals in general than between academic and community hospitals specifically. Differences in clinicians’ experience of care provision and the types of challenges faced between academic and community hospitals are unlikely to be due to differences in individual patient complexity.

## Notes

Supplement 1.
eMethods.
**eFigure 1.** Data Availability for Each Hospital **eTable 1.** GLMM Results for Baseline Clinical Characteristics **eTable 2.** GLMM Results for Social Factors at Academic and Community Hospitals **eFigure 2.** Frequency of Unique *ICD-10* Diagnosis Codes at Academic and Community Hospitals **eFigure 3.** Frequency of Unique CCSR Diagnosis Codes at Academic and Community Hospitals

Supplement 2.
Data Sharing Statement
