# Supplementary material for: Patient Complexity, Social Factors, and Hospitalization Outcomes at Academic and Community Hospitals
Source: JAMA Netw Open. 2025 Jan 15;8(1):e2454745. doi: 10.1001/jamanetworkopen.2024.54745 (PMC11736502; doi:10.1001/jamanetworkopen.2024.54745)
Supplement: Supplement 1. — eMethods. eFigure 1. Data Availability for Each Hospital eTable 1. GLMM Results for Baseline Clinical Characteristics eTable 2. GLMM Results for Social Factors at Academic and Community Hospitals eFigure 2. Frequency of Unique ICD-10 Diagnosis Codes at Academic and Community Hospitals eFigure 3. Frequency of Unique CCSR Diagnosis Codes at Academic and Community Hospitals [file jamanetwopen-e2454745-s001.pdf]

Supplemental Online Content

Colacci M, Loffler A, Roberts SB, Straus S, Vedrma AA, Razak F; GEMINI Investigator Group. Patient complexity, social factors, and hospitalization outcomes at academic and community hospitals. *JAMA Netw. Open*. 2025;8(1):e2454745. doi:10.1001/jamanetworkopen.2024.54745

eMethods.

eFigure 1. Data Availability for Each Hospital

eTable 1. GLMM Results for Baseline Clinical Characteristics

eTable 2. GLMM Results for Social Factors at Academic and Community Hospitals

eFigure 2. Frequency of Unique *ICD-10* Diagnosis Codes at Academic and Community Hospitals

eFigure 3. Frequency of Unique CCSR Diagnosis Codes at Academic and Community Hospitals

This supplemental material has been provided by the authors to give readers additional information about their work.

© 2025 Colacci M et al. *JAMA Network Open*.

eMethods.

Fitting of generalized linear mixed-effects models (GLMMs) was performed using the glmmTMB package in R. Binary and percentage response variables were modelled using binomial GLMMs. Length of stay, which was heavily right-skewed was log-transformed prior to model fitting.

Reference: Brooks ME, Kristensen K, van Benthem KJ, Magnusson A, Berg CW, Nielsen A, Skaug HJ, Maechler M, Bolker BM (2017). "glmmTMB Balances Speed and Flexibility Among Packages for Zero-inflated Generalized Linear Mixed Modeling." *The R Journal*, **9**(2), 378–400. doi:10.32614/RJ-2017-066

© 2025 Colacci M et al. *JAMA Network Open*.

eFigure 1. Data Availability for Each Hospital

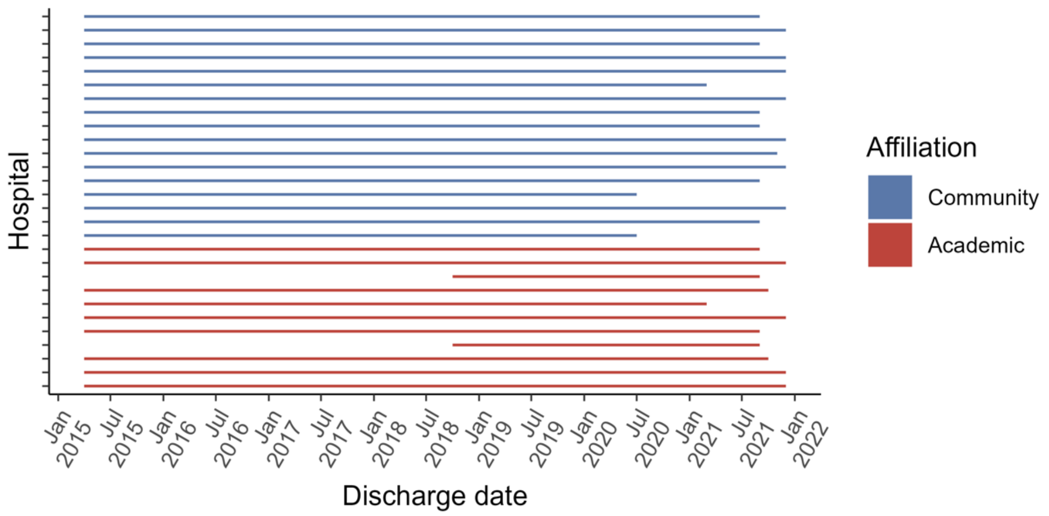

© 2025 Colacci M et al. *JAMA Network Open*.

eTable 1. GLMM Results for Baseline Clinical Characteristics

**Baseline characteristic**	$\beta_{affiliation}$/**OR**	$CI_{95\%}$	p	***ICC***	**Marginal** $R^2_{affiliation}$
Age	***B*** -2.28	[-4.50, -0.06]	0.052	0.024	0.003
Sex = male	**OR** 1.03	[0.96, 1.11]	0.421	0.003	<0.001
From long-term care home	**OR** 0.69	[0.51,0.92]	0.018*	0.044	0.009
mLAPS	***B*** 0.10	[-2.55, 2.75]	0.938	0.031	<0.001
Charlson Comorbidity Index ≥ 2	**OR** 1.01	[0.81, 1.26]	0.913	0.025	<0.001
Frailty score	***B*** 0.09	[-0.33, 0.50]	0.681	0.061	<0.001
Disability	**OR** 1.07	[0.81, 1.41]	0.624	0.038	<0.001

Legend: Beta coefficients are shown for continuous variables and odds ratios for binary variables. 95% Confidence Intervals correspond to the beta coefficient or odds ratio respectively.

© 2025 Colacci M et al. *JAMA Network Open*.

eTable 2. GLMM Results for Social Factors at Academic and Community Hospitals

**Neighborhood-level social factor**	$\boldsymbol{\beta}_{affiliation}$**/OR**	***CI*$_{95\%}$**	***p***	***ICC***	**Marginal $R^2_{affiliation}$**
Income	***β*** -1.09	[-4.58, 2.40]	0.541	0.086	0.001
Post-secondary Education (%)	**OR** 1.15	[0.95, 1.38]	0.153	0.017	0.001
Visible minorities (%)	**OR** 0.63	[0.25, 1.55]	0.310	0.304	0.011
Immigrants (%)	**OR** 0.67	[0.37, 1.21]	0.193	0.155	0.009
Deprivation	***β*** 0.05	[-0.23, 0.32]	0.733	0.101	0.000

Legend: Beta coefficients are shown for continuous variables and odds ratios for binary variables. 95% Confidence Intervals correspond to the beta coefficient or odds ratio respectively.

© 2025 Colacci M et al. *JAMA Network Open*.

eFigure 2. Frequency of unique *ICD-10* Diagnosis Codes at Academic and Community Hospitals
The horizontal dotted lines represent cut-offs at 25/50/75/90% of hospitalizations, intersected by vertical lines indicating the average number of unique ICD-10 categories that are required to reach each cut-off at community (blue) vs. academic (red) sites.

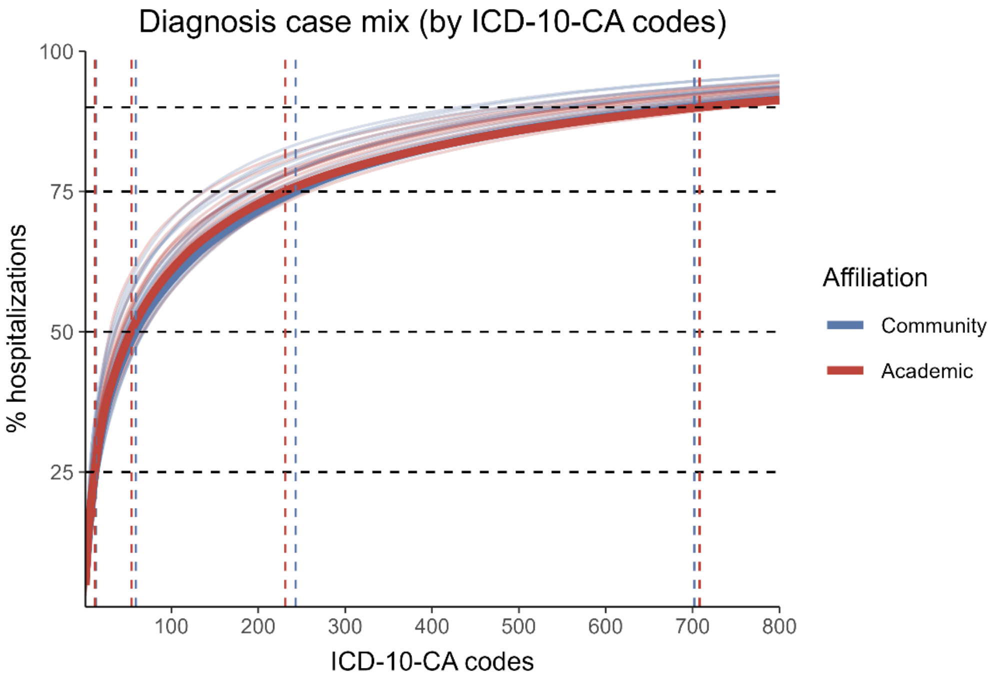

© 2025 Colacci M et al. *JAMA Network Open*.

eFigure 3. Frequency of Unique CCSR Diagnosis Codes at Academic and Community Hospitals
The horizontal dotted lines represent cut-offs at 25/50/75/90% of hospitalizations, intersected by vertical lines indicating the average number of unique CCSR categories that are required to reach each cut-off at community (blue) vs. academic (red) sites.

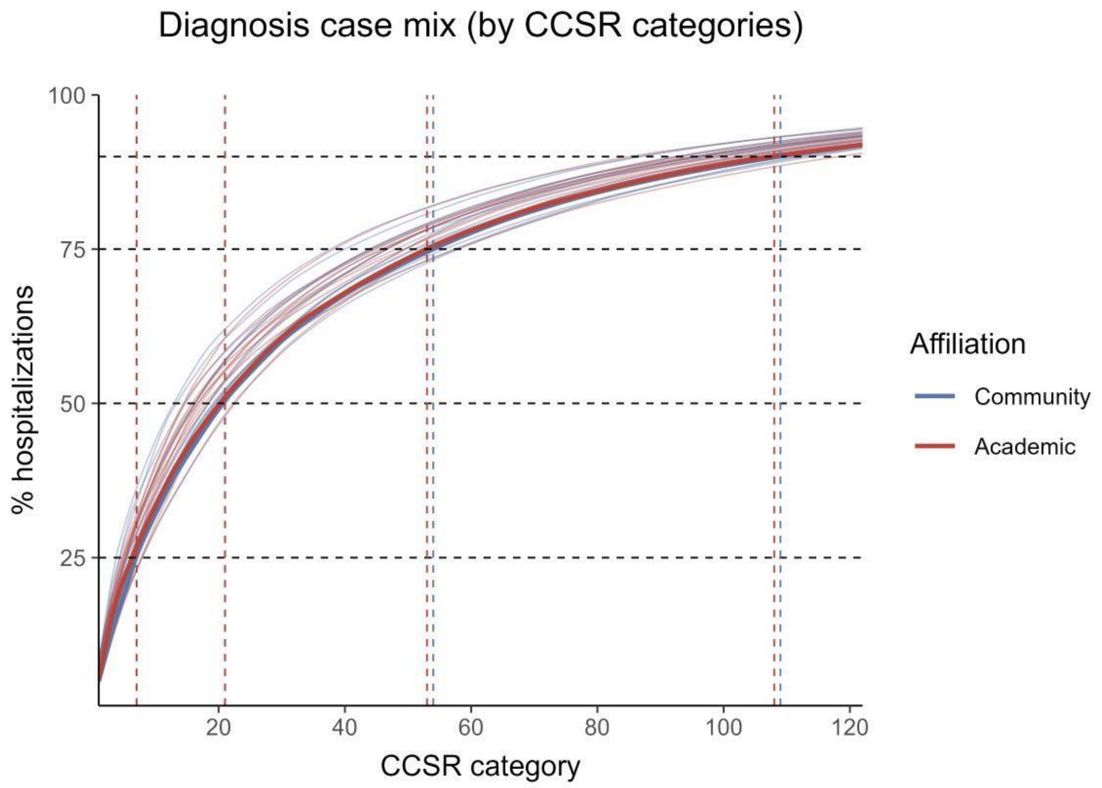

© 2025 Colacci M et al. *JAMA Network Open*.
