# Supplementary material for: Patient Complexity, Social Factors, and Hospitalization Outcomes at Academic and Community Hospitals
Source: JAMA Netw Open. 2025 Jan 15;8(1):e2454745. doi: 10.1001/jamanetworkopen.2024.54745 (PMC11736502; doi:10.1001/jamanetworkopen.2024.54745)
Supplement: Supplement 2. — Data Sharing Statement [file jamanetwopen-e2454745-s002.pdf]

Data Sharing Statement

Colacci. Patient Complexity, Social Factors, and Hospitalization Outcomes at Academic and Community Hospitals. *JAMA Netw Open*. Published January 15, 2025. doi:10.1001/jamanetworkopen.2024.54745

Data
Data available: No

Additional Information
Explanation for why data not available: The data used for this study are part of the General Medicine Inpatient Initiative (GEMINI) database. This database contains personal health information and is subject to privacy agreements and therefore cannot me made cubically available. The data can be requested by submitting a project proposal to www.geminimedicine.ca
